# Predictive Value of the Serum Albumin Level on Admission in Patients With Spontaneous Subarachnoid Hemorrhage

**DOI:** 10.3389/fsurg.2021.719226

**Published:** 2021-10-26

**Authors:** Feng Shang, Hao Zhao, Weitao Cheng, Meng Qi, Ning Wang, Xin Qu

**Affiliations:** Department of Neurosurgery, Xuanwu Hospital of Capital Medical University, Beijing, China

**Keywords:** albumin, spontaneous subarachnoid hemorrhage, Glasgow outcome scale, at admission, predictor

## Abstract

**Objective:** To determine the effect of the serum albumin level on admission in patients with spontaneous subarachnoid hemorrhage (SAH).

**Methods:** A total of 229 patients with SAH were divided into control and hypoalbuminemia groups. The serum albumin levels were measured. The data, including age, gender, co-existing medical conditions, risk factors, Hunt-Hess (H-H) grade on admission, Glasgow coma score (GCS) on admission, complications during hospitalizations, length of hospital stay, length of intensive care unit (ICU) stay, in-hospital mortality, survival rate, outcome at discharge, and the 6-month follow-up outcome, were compared between the two groups.

**Results:** Older age, an increased number of patients who consumed an excess of alcohol, and a lower GCS on admission were findings in the hypoalbuminemia group compared to the control group (*p* < 0.001). The ratio of patients with H-H grade I on admission in the hypoalbuminemia group was decreased compared to the control group (*p* < 0.05). Patients with hypoalbuminemia were more likely to be intubated, and have pneumonia and cerebral vasospasm than patients with a normal albumin level on admission (*p* < 0.001). Furthermore, the length of hospital and ICU stays were longer in the hypoalbuminemia group than the control group (*p* < 0.001). Hypoalbuminemia on admission significantly increased poor outcomes at discharge (*p* < 0.001). The number of patients with severe disability was increased and the recovery rate was decreased with respect to in-hospital outcomes in the hypoalbuminemia group than the control group (*p* < 0.001).

**Conclusion:** Hypoalbuminemia was shown to be associated with a poor prognosis in patients with SAH.

## Introduction

Subarachnoid hemorrhage (SAH) is a severe cerebrovascular disease caused by blood flowing into the subarachnoid space after intracranial vascular rupture. Subarachnoid hemorrhage is clinically divided into traumatic and non-traumatic types. Non-traumatic SAH, also known as spontaneous SAH, is a common and highly fatal stroke (1). The incidence of spontaneous SAH in China is approximately 2/100,000 per year, accounting for 5–10% of all strokes (2, 3). Intracranial aneurysms are the main cause of spontaneous SAH, accounting for approximately 85% of all cases. The clinical prognosis of SAH is relatively poor, and the mortality within 3 months of onset and the disability rate of surviving patients is also relatively high. At present, SAH is an acute hemorrhagic cerebrovascular disease that endangers human health (1, 4).

A low serum albumin level is associated with increased morbidity and mortality in various diseases (5–8). Albumin, a major component of plasma protein, has an important role in maintaining normal oncotic pressure and microvascular permeability (9–11). Hypoalbuminemia usually occurs in patients with acute ischemic stroke (12, 13). Furthermore, patients with intracerebral hemorrhage (ICH) and concurrent hypoalbuminemia are also more susceptible to poor functional outcomes (14).

There are limited data and studies that have focused on the effect of hypoalbuminemia on the outcomes of patients with SAH. Specifically, long term follow-up studies with a large number of patients have been insufficient (15–20). Therefore, we determined the influence of a low serum albumin level at the time of admission in patients with spontaneous SAH.

## Subjects and Methods

### Subjects

This retrospective study was approved by the Hospital Review Board. A total of 229 patients who were admitted to our Neurosurgery intensive care unit (ICU) with a diagnosis of spontaneous SAH from December 2017 to December 2018 were enrolled. The diagnosis of SAH met the diagnostic criteria of the 2015 edition of Chinese Guidelines for the Diagnosis and Treatment of Subarachnoid Hemorrhage (2). The inclusion criteria were as follows: (1) spontaneous onset; (2) the diagnostic criteria for SAH were met; (3) hospitalized within 7 days of onset; (4) serum albumin levels were closely monitored within 24 h after admission; (5) a head CT, lumbar puncture, and head and neck CTA/DSA/MRA examinations obtained after admission; and (6) complete medical records. The exclusion criteria were as follows: (1) died within 24 h after admission; (2) traumatic or spontaneous SAH undergoing craniotomy; (3) heart, lung, liver, kidney, and other vital organ dysfunction; (4) a history of severe coagulation dysfunction; (5) incomplete medical records; and (6) no serum albumin level obtained on admission.

### Determination of Serum Albumin Levels

The serum albumin levels were measured at 24 h and 1 week after onset. The serum albumin level was commonly measured by recording a change in absorbance upon binding to a dye, such as bromocresol green or bromocresol purple (21, 22). Hypoalbuminemia was established when the serum albumin level was <3.5 g/dl (23). Patients were divided into control and hypoalbuminemia groups depending on the diagnosis of hypoalbuminemia on admission.

### Data Collection

The baseline demographics (age and gender), other diseases (diabetes mellitus, hyperlipidemia, and coronary artery disease), risk factors [hypertension, smoking, and excessive alcohol consumption (24)], admission Hunt-Hess (H-H) grade (25), Glasgow coma score (GCS) on admission, complications during hospitalization [intubation, pneumonia, cerebral vasospasm, re-bleeding, and delayed cerebral ischemia (DCI)], length of hospital stay, length of ICU stay, in-hospital mortality, survival rate, outcomes at discharge, and 6-month follow-up outcomes were collected. Hypertension was diagnosed if the systolic blood pressure was >140 mmHg or the diastolic blood pressure was >90 mmHg, or the patient was taking anti-hypertensive medications. Cerebral vasospasm was described as focal or diffuse temporarily narrowed vessel caliber due to the contraction of a smooth muscle in the wall of the arteries, which was detected by angiography, transcranial Doppler (TCD), magnetic resonance (MR), and CT (26). Rebleeding was identified as an acute clinical deterioration that was accompanied by evidence of rebleeding in the subarachnoid space, ventricular system, or brain parenchyma via a follow-up CT or an autopsy (27). Delayed cerebral ischemia (DCI) was defined as the development of new focal neurologic signs and/or a deterioration in the level of consciousness lasting for >1 h, or the appearance of new infarctions on CT or MRI (28–30).

### Outcome Measures

Poor outcome was defined as in-hospital death, or transfer to hospice care or a nursing home facility [GOS scale ≤ 3 (27)]. Serious complications included another or several potentially fatal diseases triggered during the course of the SAH. Furthermore, the 6-month follow-up outcomes were also recorded.

### Statistical Analysis

Statistical analysis was performed by SPSS17.0 (International Business Machines, Corp., Armonk, NY, USA). The differences between the two groups were compared by a chi-square test or Fisher's exact test for categorical data and Student's *t*-test or Kruskal test for continuous data. Multivariable logistic or linear regression analysis was carried out to study the relationship between the albumin level and outcomes. All the multivariable regression models were repeated for the characteristic variables. A *p*-value ≤ 0.05 was considered statistically different.

## Results

### General Clinical Characteristics

Three hundred and fifteen patients with spontaneous SAH were admitted in the study. Among the 315 patients, 229 met the inclusion criteria, including 79 with normal albumin levels and 150 with hypoalbuminemia. The albumin level in the hypoalbuminemia group was decreased more than the control group (*p* < 0.001). The baseline data are shown in Table 1. The age in the hypoalbuminemia group was higher than the control group (*p* < 0.001). The number of patients that consumed excessive alcohol was greater and the mean GOS on admission was lower in the hypoalbuminemia group than the normal albumin group (*p* < 0.001). Moreover, the ratio of patients with H-H grade I on admission in the hypoalbuminemia group was lower than the control group (19.3 vs. 38%, *p* < 0.05). Furthermore, the ratio of patients with H-H grade III on admission was higher in the hypoalbuminemia group than the control group (19.3 vs. 5.1%, *p* < 0.05).

**Table 1:** Baseline characteristics of patients with spontaneous subarachnoid hemorrhage.

**Items**	**All patients** **(*n* = 229)**	**Low serum albumin** **(*n* = 150)**	**Normal serum albumin** **(*n* = 79)**	**Test method**	**Test statistic**	***p*-Values**
Age (years)	55.53 ± 13.11	58.84 ± 11.06	49.24 ± 14.39	Kruskal	22.68	0.001^*^
Gender (Males)	103 (45.0%)	61 (40.7%)	42 (53.2%)	Chi square	3.27	0.071
Admission H-H grade				Chi square	15.50	0.001^*^
I	59 (25.8%)	29 (19.3%)	30 (38%)	Chi square	3.52	0.002^*^
II	96 (41.9%)	57 (38.0%)	39 (49.4%)	Chi square	2.75	0.097
III	33 (14.4%)	29 (19.3%)	4 (5.1%)	Chi square	8.54	0.003^*^
IV	26 (11.4%)	20 (13.3%)	6 (7.6%)	Chi square	1.69	0.193
V	5 (2.2%)	5 (3.3%)	0 (0%)	Chi square	2.69	0.244
Hypertension	129 (56.3%)	82 (54.7%)	47 (59.5%)	Chi square	0.49	0.484
Smoking	90 (39.3%)	64 (42.7%)	26 (32.9%)	Chi square	2.06	0.151
Alcohol consumption	49 (21.4%)	47 (31.3%)	2 (2.5%)	Chi square	25.52	0.001^*^
Diabetes mellitus	18 (7.9%)	11 (7.3%)	7 (8.9%)	Chi square	0.17	0.683
Hyperlipidemia	6 (2.6%)	2 (1.3%)	4 (5.1%)	Chi square	2.82	0.213
Coronary artery disease	15 (6.6%)	13 (8.7%)	2 (2.5%)	Chi square	3.18	0.074
Admission Glasgow coma score	13.34 ± 3.31	12.86 ± 3.65	14.25 ± 2.28	Kruskal	15.45	<0.001^*^

*Measurement data are expressed as means ± standard deviation (SD) and counting data are expressed as a percentage*.

* *p ≤ 0.05: low serum albumin group vs. normal serum albumin group. H-H grade, Hunt-Hess grade*.

### Comparison of Prognosis Between the Two Groups

Patients with hypoalbuminemia were more likely to be intubated, and have pneumonia and cerebral vasospasm than patients with a normal albumin level on admission (43.6 vs. 8.9%, *p* < 0.001; 46.3 vs. 13.9%, *p* < 0.001; and 50.7 vs. 32.9%, *p* = 0.010, respectively; Table 2). In addition, the length of hospital and ICU stays were longer in the hypoalbuminemia group than the control group (13.1 vs. 4.8 days, *p* < 0.001; and 12.2 vs. 4.6 days, *p* < 0.001, respectively). There was a significant increase in poor outcomes at the time of hospital discharge in the hypoalbuminemia group than the control group (30.0 vs. 2.5%, *p* < 0.001; OR = 33.4; 95% CI: 8.4–132.8). Considering the outcomes at the time of hospital discharge, the severe disability and recovery rates were significantly different between the two groups (*p* < 0.001), and this disparity persisted to the 6-month follow-up outcomes (*p* = 0.045; *p* = 0.032). The leading causes of death during hospitalization and at the 6-month follow-up included severe primary diseases (mainly with severe and high grade SAH) and severe complications (rebleeding, severe cerebral vasospasm, hydrocephalus, epilepsy, and cerebral hernia). Fourteen patients were lost to follow-up by the 6-month evaluation. The number of patients decreased from 229 at the beginning of the study to 215 after follow-up.

**Table 2:** Correlation of serum albumin levels in patients with spontaneous subarachnoid hemorrhage.

**Items**	**All** **(*n* = 229)**	**Low serum albumin** **(*n* = 150)**	**Normal serum albumin** **(*n* = 79)**	**Test method**	**Test statistic**	***p*-Values**
Intubation	72 (31.6%)	65 (43.6%)	7 (8.9%)	Chi square	28.88	<0.001^*^
Pneumonia	80 (35.1%)	69 (46.3%)	11 (13.9%)	Chi square	23.77	<0.001^*^
Cerebral vasospasm	102 (44.5%)	76 (50.7%)	26 (32.9%)	Chi square	6.60	0.010^*^
Delayed cerebra infarction	40 (17.5%)	27 (18.0%)	13 (16.5%)	Chi square	0.09	0.770
Rebleeding	18 (7.9%)	15 (10.1%)	3 (3.8%)	Chi square	2.79	0.095
Length of hospital stay (days, median)	10.19 ± 10.42	13.05 ± 11.34	4.76 ± 5.07	Kruskal	53.54	0.001^*^
Length of ICU stay (days)	9.54 ± 10.36	12.15 ± 11.44	4.60 ± 5.11	Kruskal	42.77	0.001^*^
Poor outcome	46 (20.1%)	45 (30.0%)	2 (2.5%)	Chi square	23.94	0.001^*^
Discharge outcome				Chi square	32.26	0.001^*^
Death	4 (1.7%)	4 (2.7%)	0 (0%)	Chi square	2.14	0.350
Vegetative stage	10 (4.4%)	9 (6.0%)	1 (1.3%)	Chi square	2.78	0.185
Severe disability	32 (14.0%)	32 (21.3%)	1 (1.3%)	Chi square	16.90	0.001^*^
Mild disability	39 (17.0%)	30 (20.0%)	9 (11.4%)	Chi square	2.71	0.100
Recovery	144 (62.9%)	75 (50.0%)	68 (86.1%)	Chi square	28.72	0.001^*^
6-month follow-up outcome				Chi square	10.38	0.040^*^
Death	25 (10.9%)	15 (10.0%)	5 (6.3%)	Chi square	1.17	0.280
Vegetative stage	2 (0.9%)	2 (1.3%)	0 (0%)	Fisher	-	0.546
Severe disability	10 (4.4%)	10 (6.7%)	0 (0%)	Chi square	5.51	0.045^*^
Mild disability	22 (9.6%)	14 (9.3%)	13 (16.5%)	Chi square	2.52	0.112
Recovery	156 (68.1%)	95 (63.3%)	61 (77.2%)	Chi square	4.59	0.032^*^

*Measurement data are expressed as means ± standard deviation (SD) and counting data are expressed as a percentage*.

* *p ≤ 0.05: low serum albumin group vs. normal serum albumin group. ICU, intensive care unit*.

### Independent Predictors

After adjusting for age, gender, H-H grade on admission, excessive alcohol consumption, coronary artery disease, and GCS on admission, we showed that hypoalbuminemia (OR = 4.7, *p* = 0.006), pneumonia (OR = 2.6, *p* = 0.024), poor outcome (OR = 18.51, *p* = 0.006), length of hospital stay (effect size: 5.33 days, *p* < 0.001), and length of ICU stay (effect size: 4.29 days, *p* = 0.001) were independent predictors for intubation (Table 3).

**Table 3:** Multivariable analysis of serum albumin levels in patients with spontaneous subarachnoid hemorrhage.

**Items**	**Low serum albumin**	
	**OR (95%CI)**	***p*-Values**
Intubation	4.7 (1.67–16)	0.006^*^
Pneumonia	2.6 (1.16–6.2)	0.024^*^
Vasospasm	1.75 (0.9–3.45)	0.100
Cerebral infarction	0.97 (0.4–2.41)	0.954
Rebreeding	3.77 (0.82–28.05)	0.124
Poor outcome	18.51 (3.41–349.03)	0.006^*^
	**β (95%CI)**	***p*** **-Values**
Length of hospital stay	5.33(2.81–7.85)	0.001^*^
Length of ICU stay	4.29(1.83–6.74)	0.001^*^

* *p ≤ 0.05*.

## Discussion

A low serum albumin level induces numerous health issues (5–8). The predictors of mortality and morbidity in patients with spontaneous SHA include age, hypertension, low GCS on admission, cerebral vasospasm, rebleeding, and DCI (24–29). In our study, most of these predictors, except for hypertension and DCI, were very different between the two groups, indicating that a multiple logistic regression analysis may be needed to further assess the correlation between hypoalbuminemia and outcomes at discharge.

The serum albumin level is a function of rates of synthesis, degradation, and distribution in the extracellular and intracellular spaces (9). Hypoalbuminemia may occur not only in patients with liver or kidney dysfunction and malnutrition, but also in the distribution or catabolism and the presence of inflammatory cytokines (31).

Only two-thirds of men 65–74 years of age with serum albumin levels ≥4.4 g/dl have developed strokes compared to patients with albumin levels ≤4.2 g/dl (32). The prevalence of renal dysfunction has been reported in 20–35% of patients with ischemic strokes and 30–46% of patients with ICH (33). Therefore, hypoalbuminemia may in part be a result of complex cerebrorenal interactions and renal dysfunction (34–36). A low albumin level could be a modifiable factor for patients with spontaneous SAH or other conditions.

There should be no major issue with the use of albumin in most developed countries and more advanced developing countries; however, for most low- and middle-income countries (LMICs), it is more difficult to assess serum chemistries, such as albumin, when it is even difficult to meet the requirements of essential medications and medical equipment (Is this change ok?). These issues are really difficult to overcome, and can only be handled according to the actual situation of the respective hospital departments.

There were some limitations in our study. The main limitation was the retrospective nature of the study. The simple laboratory test is needed to assess this alteration in the albumin level. Moreover, this was a single center study with a small sample size. The retrospective nature of data collection, the lack of information on the premorbid nutritional and volume status, the absence of serial albumin measurements, and long-term functional outcomes are all limitations. Larger prospective studies with serial measurements of serum albumin levels are needed. Therefore, the role of albumin supplementation in patients with spontaneous SAH are warranted.

## Conclusion

In conclusion, we demonstrated that hypoalbuminemia on admission is a risk factor for poor outcome in patients with spontaneous SAH. It is substantially relevant for prediction and prognosis of spontaneous SAH. Because measuring the serum albumin level is an affordable task, it may serve as an additional prediction marker in these patients. Larger prospective studies with serial measurements of serum albumin are required to identify the poor outcomes and thereby implement therapeutic interventions.

## Data Availability Statement

The raw data supporting the conclusions of this article will be made available by the authors, without undue reservation.

## Ethics Statement

The studies involving human participants were reviewed and approved by Xuanwu Hospital, Capital Medical University Committee. The patients/participants provided their written informed consent to participate in this study. Written informed consent was obtained from the individual(s) for the publication of any potentially identifiable images or data included in this article.

## Author Contributions

FS and HZ: designed the project. FS, HZ, WC, MQ, and NW: were responsible for experiments data collection and analysis, and manuscript writing. XQ revised the manuscript. All the authors participated in the review of the manuscript.

## Conflict of Interest

The authors declare that the research was conducted in the absence of any commercial or financial relationships that could be construed as a potential conflict of interest.

## Publisher's Note

All claims expressed in this article are solely those of the authors and do not necessarily represent those of their affiliated organizations, or those of the publisher, the editors and the reviewers. Any product that may be evaluated in this article, or claim that may be made by its manufacturer, is not guaranteed or endorsed by the publisher.
